# Accuracy of Race and Ethnicity Data in the Pediatric Electronic Health Record: A Concordance and System Adequacy Study

**DOI:** 10.1089/heq.2024.0188

**Published:** 2025-05-12

**Authors:** John D. Cowden, Rachel Drake, Jessi Johnson, Katiana Kelty, Mehwish Ahmed

**Affiliations:** ^1^ Department of Pediatrics, Children’s Mercy Kansas City, Kansas City, Missouri, USA.; ^2^ University of Missouri Kansas City—School of Medicine, Kansas City, Missouri, USA.; ^3^ Division of Urgent Care, Children’s Mercy Kansas City, Kansas City, Missouri, USA.; ^4^ Office of Equity and Diversity, Children’s Mercy Kansas City, Kansas City, Missouri, USA.; ^5^ Kansas City University College of Osteopathic Medicine, Kansas City, Missouri, USA.

**Keywords:** race and ethnicity, information technology, electronic health record, data accuracy

## Abstract

**Introduction:**

Conventional race and ethnicity categories and analysis are reductive and prone to inaccuracy. Because race and ethnicity data validity is essential to health equity efforts, we measured the accuracy of race and ethnicity data in a pediatric electronic health record (EHR) to identify areas for improvement in data collection and use.

**Methods:**

Patients and their caregivers reported patient race and ethnicity via in-person survey in four pediatric settings (inpatient, emergency room, urgent care, and primary care). Race and ethnicity data from the EHR were compared with survey data to calculate four measures of EHR data accuracy. The U.S. Census Bureau’s novel categorization scheme was used to analyze racial and ethnic identities “alone” and “in combination” with ≥1 other identity.

**Results:**

Caregivers for 561 patients completed the survey; 116 patients aged ≥12 years completed a patient version. For consolidated race and ethnicity fields, overall concordance between survey and EHR was 74.6%. Concordance differed by race and ethnicity category when alone (Black or African American 96.1%, Hispanic 90.6%, and White 92.5%) and in combination with another category (Black or African American 93.9%, Hispanic 88.6%, and White 84.4%). The EHR had low accuracy for patients with multiple racial or ethnic identities (overall sensitivity 35%). Such patients’ identities were often oversimplified due to EHR design. Using “alone” and “in combination” analysis for race and ethnicity categories allowed all patient identities to be visible across categories, unlike in conventional race and ethnicity analysis.

**Discussion:**

Identifying and eliminating health disparities depend on accurate race and ethnicity data, but current EHR design provides an unreliable data foundation for needed analyses. Conventional categorization used in race and ethnicity analysis is problematic, hiding identities in a reductive set of groupings. New approaches to validation, categorization, and analysis, as explored in this study, are urgently needed to advance health equity goals.

## Introduction

The U.S. population is increasingly diverse, with nearly 40% of all Americans and over half of those under 16 years old identifying with a racial or ethnic group other than White.^1^ The proportion of those identifying with more than one race or ethnicity is also rising, especially in younger age groups. Between 2010 and 2020, the number of Americans identifying as having multiple races more than doubled among non-Hispanics and rose over sixfold among Hispanics.^2^ Factors contributing to this increase include the birth of more multiracial babies, individuals rethinking their identities, and new ways to collect and analyze detailed race and ethnicity data.^2^

As diversity has increased, so has awareness of racial and ethnic health disparities.^3^ Accurate race and ethnicity data collection is central to addressing these health disparities, as well as identifying changes in population health and evaluating treatment plans and effectiveness in pediatric patients.^4^ Accurate data are also crucial to developing interventions that address the needs of different population groups. The importance of accurate data is highlighted in best practice guidance for identifying and addressing disparities,^5^ where validation of existing race and ethnicity data is included as a foundational step before disparities analysis. Using underlying data without understanding its validity can lead to mistaken conclusions and misguided action.

Conventional race and ethnicity data collection in health care settings has not kept pace with the changes in the U.S. Census that have led to a more detailed understanding of Americans’ identities.^4^ Current electronic health record (EHR) systems often include race and ethnicity data fields and options reflecting the minimum standard defined in 1997 by the U.S. Federal Government.^6^ As a result, the data fields and categories available for patients in EHRs are typically reductive and more limited than the emerging best practices for capturing racial and ethnic identity.^7^ On top of this, the concepts of race and ethnicity are complex and subjective, creating potentially challenging discussions between staff and patients/families in health care settings, such as during patient registration. Although it is the best practice to ask patients or their parents/caregivers to self-report race and ethnicity, quality improvement work at our institution has shown that sometimes patient registration staff assume a person’s identity rather than asking a potentially uncomfortable question.

Concern about the accuracy and limitations of race and ethnicity data in health records has been raised by multiple studies showing variable concordance between data in the EHR and a gold standard (e.g., direct reporting by patient to researchers).^8–12^ At our health care organization, we recognized that (1) our growing health equity efforts require us to understand whether our EHR might have similar inaccuracy and (2) the structure of race and ethnicity data fields and categories likely need updating to adequately reflect patients’ true identities. To validate our existing race and ethnicity data and evaluate the adequacy of our current data collection system, we designed this descriptive study to compare EHR data with direct report of racial and ethnic identities by patients and their parents or other caregivers.

## Methods

### Study design and participants

We conducted a cross-sectional concordance study comparing race and ethnicity data collected by survey in person from a convenience sample of patients and their caregivers (the “gold standard”) to existing race and ethnicity data in the pediatric EHR for the same patients. Participants included caregivers of patients 0–17 years old and patients ≥12 years old at a large pediatric health system providing comprehensive clinical services. The pediatric health system’s catchment area includes a metropolitan area of ∼2 million people and the surrounding four-state area. This study was approved by the Children’s Mercy Hospital Institutional Review Board.

### Data collection

Participants were recruited from four clinical sites over 4 months (November 2021–February 2022): inpatient wards, emergency room, urgent care clinic, and primary care clinic. Inclusion criteria included patients of any age at one of the four study sites accompanied by at least one parent or other legal guardian. All languages were included. Exclusion criteria included positive coronavirus disease (COVID) status (to minimize the risk of spreading COVID) or a clinical team request not to recruit due to patient or family circumstance.

#### Survey instrument

We developed an electronic survey using Research Electronic Data Capture (REDCap)^13^ to gather self-reported race and ethnicity from patients and caregivers. Between 16 and 28 questions were included in each interaction depending on patient age, number of primary caregivers present, and branching logic. An initial version based on existing hospital race and ethnicity questions, published best practices,^14^ and study team expertise was piloted with 20 families who provided survey feedback in addition to responding to survey items. We used feedback from participants and surveyors to create the final survey (Appendix).

Survey sections reported in this article addressed (1) demographics (patient age, study site, caregiver type), (2) patient report (≥12 years) of own Hispanic identity (yes/no and granular) and other races and ethnicities (free answer, multiple allowed), and (3) caregiver report of patient’s Hispanic identity and other races and ethnicities (free answer, multiple allowed).

#### Survey procedure

Three surveyors (R.D., K.K., and a research coordinator) were trained in survey implementation and recruited patients and their caregivers at each study site. To assure an adequate sample of non-English-speaking caregivers, we oversampled in the outpatient settings, where there is a higher proportion of families preferring a language other than English. Survey sessions occurred at various times of day on weekdays and weekends. The surveyor entered the patient’s room and explained the purpose of the study, providing an information sheet in English, Spanish, Arabic, Vietnamese, or Somali (for other languages, the surveyor read the English sheet using a video interpreter). After obtaining verbal consent from the caregiver(s) or patient over 18 and assent from younger patients, the survey was read aloud by the surveyor to the participants (using a video interpreter when appropriate). Responses were recorded by the surveyor via REDCap on an electronic tablet. No incentives were offered to participants.

#### EHR data

A data analyst created a report of EHR race and ethnicity data that we matched to survey response data using medical record numbers. EHR data had been previously entered by patient registration staff during routine patient intake the first time a patient presented for care at our institution (not necessarily the visit when the family was approached for this study). In the course of this study, patient registration staff were not aware that families might be approached later in their visit to take part in a study on race and ethnicity data accuracy. The final unified data set included five fields from the EHR (Hispanic ethnicity, race, free-text race [for “multiracial” or “other”], granular ethnicity [any further ethnic identities offered by respondent], and free-text granular ethnicity [for “multiethnic” or “other”]) and survey data described above.

### Data analysis

We compared patient race and ethnicity data from the EHR with those from the survey to calculate overall EHR accuracy (# patients correctly assigned/total # patients) and intermeasure reliability (Cohen’s kappa statistic). Comparison of specific race and ethnicity categories was made using the chi-square analysis (with Yates’ correction for expected cell count <5). We calculated accuracy, sensitivity, and specificity for each race and ethnicity category (alone and in combination with at least one other race or ethnicity).

For analysis of overall EHR accuracy and intermeasure reliability, we used single patient race and ethnicity values from each data set. For teen patients in the survey data set, we used the caregiver response (rather than the teen’s) for comparison with the EHR because teens are not asked their race and ethnicity when registering at our institution—only their caregivers are asked. In the two cases when caregivers disagreed on the patient’s race and ethnicity, we used the response from the caregiver most likely to bring the patient for care. When more than one racial or ethnic identity was reported in either the EHR or survey, the term “multiracial” was used as the single value. For category-specific analysis, individual race and ethnicity identities included American Indian or Alaska Native, Asian, Black or African American, Hispanic, Middle Eastern or North African, Multiracial, Native Hawaiian, or Other Pacific Islander, and White. Other words used for race or ethnicity were rolled into these categories according to guidance from the Institutes of Medicine.^15^ A race and ethnicity identity was considered “alone” when a single word or multiple near-synonymous words were reported. The identity was considered “in combination” when two or more nonsynonymous words were reported.

## Results

Caregivers for 561/601 patients (93%) completed the survey; 116 patients ≥12 years old completed the patient version. Survey location and participant demographics are shown in Table 1.

**Table 1.** Survey Locations and Demographics of Study Participants at Children’s Mercy Kansas City, 2021–2022

	No. (%)
Location (*n* = 561)	
Pediatric primary care clinic (offsite)	196 (34.9)
Pediatric urgent care (offsite)	136 (24.2)
Pediatric emergency department	129 (23.0)
Inpatient medical/surgical units	100 (17.8)
Caregiver (*n* = 682)	
Mother	488 (71.6)
Father	172 (25.2)
Grandmother	9 (1.3)
Other	13 (1.9)
Patient (*n* = 561)	
Age	
<1 year	161 (28.7)
1–4 years	137 (24.4)
5–11 years	147 (26.2)
12–17 years	108 (19.3)
≥18 years	8 (1.4)

For some patients, multiple caregivers responded, making the number of caregiver participants (*n* = 682) larger than the number of patient participants (561). Most caregivers were mothers (71.6%) or fathers (25.2%). Patients from the pediatric primary care clinic and infants were slightly more common in the sample due to the oversampling described above.

### Accuracy

The distribution of race and ethnicity categories in the survey and EHR, both alone and in combination with at least one other category, are shown in Table 2.

**Table 2.** Race and Ethnicity of Patient Participants (*n* = 561) at Children’s Mercy Kansas City, 2021–2022

	Survey no. (%)	EHR no. (%)	*p*-Value^a^
American Indian or Alaska Native			0.25
Alone	0 (0)	1 (0.2)	
In combination	42 (7.5)	5 (0.9)	
Alone or in combination	42 (7.5)	6 (1.1)	
Asian			0.23
Alone	11 (2.0)	8 (1.5)	
In combination	8 (1.4)	2 (0.4)	
Alone or in combination	19 (3.4)	10 (1.8)	
Black or African American			**0.006**
Alone	86 (15.4)	100 (18.4)	
In combination	66 (11.8)	39 (7.2)	
Alone or in combination	152 (27.2)	139 (25.6)	
Hispanic or Latino			**<0.001**
Alone	166 (29.7)	201 (36.9)	
In combination	82 (14.7)	36 (6.6)	
Alone or in combination	248 (44.4)	237 (43.6)	
Middle Eastern or North African			0.40
Alone	1 (0.2)	0 (0)	
In combination	3 (0.5)	1 (0.2)	
Alone or in combination	4 (0.7)	1 (0.2)	
Native Hawaiian or Other Pacific Islander			0.74
Alone	5 (0.9)	4 (0.7)	
In combination	0 (0)	0 (0)	
Alone or in combination	5 (0.9)	4 (0.7)	
White			**<0.001**
Alone	142 (25.4)	167 (30.7)	
In combination	115 (20.6)	48 (8.8)	
Alone or in combination	257 (46.0)	215 (39.5)	
Number of Races and/or Ethnicities			**<0.001**
1	411 (73.3)	481 (85.7)	
≥2	150 (26.8)	63 (11.6)	
Unknown^b^	0 (0)	17 (3.0)	

Bold font indicates p-Value <0.05.

^a^ Chi-square analysis, with Yates’ correction when expected frequency <5 for any cell.

^b^ Unknown category includes “unknown to respondent,” “respondent not available,” and “declined/refused.”

EHR, electronic health record.

Black or African American, Hispanic, and White identities were significantly more likely to be reported in combination with another identity in the survey than in the EHR (11.8% vs. 7.2%, *p* = 0.006; 14.7% vs. 6.6%, *p* < 0.001; 20.6% vs. 8.8%, *p* < 0.001, respectively). The overall proportion of patients with multiple racial and ethnic identities was also significantly higher in the survey than in the EHR (26.8% vs. 11.6%, *p* < 0.001).

Overall concordance for race and ethnicity (# patients correctly assigned/total # patients) between survey and EHR was 76.8%. Analysis of intermeasure reliability yielded a kappa statistic of 0.72 (95% confidence interval: 0.68–0.76; standard error [SE] 0.02), characterized as “good” agreement according to Altman.^16^ Table 3 shows measures of accuracy for the race and ethnicity categories with sample sizes large enough to derive reliable measures (i.e., Black or African American, Hispanic, White).

**Table 3.** Accuracy of Electronic Health Record (EHR) Race and Ethnicity Data for Pediatric Patients in One or More of Four Statistically Analyzable Categories (*N* = 531)^a^

	Concordance (%)	Sensitivity (%)	Specificity (%)
Black or African American			
Alone (*n* = 100)^b^	96.1	97.6	95.9
In combination (*n* = 39)	93.9	54.8	99.0
Alone or in combination (*n* = 139)	96.3	91.0	98.2
Hispanic			
Alone (*n* = 167)	90.6	94.0	89.2
In combination (*n* = 36)	88.6	33.3	97.9
Alone or in combination (*n* = 233)	94.7	91.8	97.0
White			
Alone (*n* = 167)	92.5	95.7	91.4
In combination (*n* = 48)	84.4	33.0	97.2
Alone or in combination (*n* = 215)	87.5	79.8	93.9

^a^ All other categories had sample sizes too small to derive reliable accuracy statistics.

^b^ Sample sizes for each race and ethnicity category from EHR.

Concordance ranged from 84.4% to 96.3% in these categories, with highest concordance numbers for Black or African American and lowest for White. The EHR showed notable weakness in identifying patients with multiple racial and/or ethnic identities, with low sensitivity numbers for the “in combination” groups for all three categories (Black or African American 54.8%, Hispanic 33.3%, and White 33.0%). This means that when these identities were true in real life (i.e., by survey response), the EHR picked them up accurately only between about 1/3 and 1/2 the time. In contrast, specificity was high for all three, meaning that when a patient did not have multiple racial and/or ethnic identities, the EHR almost always showed that to be true.

### Teen–caregiver and caregiver–caregiver agreement

A total of 106/108 (98%) teen patients from 12 to 17 years old were surveyed. Ten (9.4%) said they were unsure of their racial and ethnic identity. For the 96 teens reporting one or more identities, caregivers reported something different for 18 (19%), adding more precise, more general, or similar identities (e.g., teen said “Mexican,” caregiver added “Hispanic”). Occasionally, a caregiver reported an identity the teen was not aware of. When multiple caregivers were present (*n* = 132), there was disagreement on the race and ethnicity of the patient in two (1.5%) cases.

## Discussion

We found that the accuracy of EHR race and ethnicity data in a large pediatric health system depended on the race and ethnicity category, with notable weakness in reflecting multiracial and multiethnic identities. Our findings are consistent with race and ethnicity data accuracy studies of adult patients,^8–12^ especially in showing accuracy varies by category. As in our study, Proumen and others found that all 117 adult respondents self-identifying as multiracial were misidentified in an EHR.^12^ As far as we could find, ours is the first study of race and ethnicity data accuracy in a pediatric health system.

The meaning of race and ethnicity data accuracy estimates depends on how the data are used and the level of tolerance for inaccuracy that comes with that use. When used for descriptive demographics, inaccuracy can be shown using confidence intervals to give an idea of how far off estimates might be. When used for complex analysis, though, inaccuracy is more problematic. Race and ethnicity data are commonly used for stratified analysis of health care processes and outcomes, looking for possible racial and ethnic disparities in measures such as hospital length of stay, disease outcomes, and mortality. In such analyses, inaccuracy in the primary exposure variable (race and ethnicity) at the level shown in our study (i.e., 76.8% overall concordance, Cohen’s kappa 0.72) could lead to false conclusions about the absence/presence and magnitude of disparities. The direction of such errors is difficult to predict, since patients with multiple racial and/or ethnic identities may be reductively classified into either advantaged or disadvantaged monoracial groups depending on the cause of and motive for such classification.^17,18^ While this could hide existing disparities or reveal false ones, it also ignores the unique experiences of multiracial/ethnic individuals, a problem recently gaining overdue attention in the health literature.^19,20^ With disparities analyses increasingly expected to be part of health care organizations’ standard quality improvement processes,^21,22^ understanding the accuracy of demographic variables such as race and ethnicity is essential for confident and meaningful analysis of health equity-related outcomes.

In our survey, questions about race and ethnicity were similar to those used during registration in our organization, but with important additions. After each question about Hispanic identity and racial identity, the survey respondent was asked if there were any other words they would use to describe the child’s (or for teen patients their own) heritage, background, or ancestry. For many patients, the additional identities were not synonymous with an identity already given, leading to identities being offered that did not appear in the EHR. This led to an increased likelihood to be in race and ethnicity categories “in combination” with another identity rather than “alone.” In a 2021 survey study of a representative group of ∼3500 U.S. residents, the Pew Research Center found that only about half (49%) of residents felt the 2020 Census race and ethnicity questions reflected their identity “very well.”^23^ Our respondents’ behavior, in going beyond simple, traditional self-identifiers, likewise supports the idea that our current approach to asking about race and ethnicity is inadequate for fully capturing patient identities.

Differences in race and ethnicity data between the EHR and our survey are likely related to question and data field design, with EHR fields and responses based on best practice from 2010, while the survey incorporated current best practice to detect all aspects of a patient’s racial and ethnic identity (representing the gold standard). We found the design of our organization’s EHR, which includes three fields related to race and ethnicity: (1) “Ethnicity,” (2) “Race,” and (3) “Nationality,” to be problematic for data collection. One author (R.D.) interviewed registration staff about the use of these fields, finding that the names of the fields in the EHR were sometimes confusing for staff and families (e.g., “Ethnicity” only including a Hispanic option and “Nationality” not meant to represent someone’s legal nationality, but their ethnic background[s]) and that the “Nationality” question was not always asked because it is not required (unlike the Hispanic ethnicity and race questions). This confusion and variability in collection may have contributed to the inaccuracy in race and ethnicity data in our EHR. Our survey also included three questions, but the wording was nuanced, providing the opportunity to report all the words used to describe a patient’s race, ethnicity, heritage, background, or ancestry. We avoided the term “nationality,” instead asking after each main question whether there were any other words that the respondent used to describe the patient’s heritage, background, or ancestry.

The approach to analysis used in our study is taken from the U.S. Census Bureau,^24^ which introduced the “in combination,” “alone,” and “in combination or alone” method for studying the most recent 2020 Census data. The new approach was adopted due to problems inherent to conventional racial and ethnic groupings in the United States that simplify identity into the categories “Hispanic,” “Black” (sometimes “non-Hispanic Black”), “White” (sometimes “non-Hispanic White”), and lumped groups such as “multiracial” and “other.” The primary problem in this approach is that the details of identity are hidden by reductive terminology and categorization, prohibiting an individual from being seen in the analysis as both Black and Hispanic, for example. Disparities analysis based on such arbitrarily simplified groups may lead to conclusions about health care processes and outcomes that are fundamentally flawed, since individuals with multiple identities are miscategorized as having a single identity. Our use of the newer approach led to a more detailed summary of identities and a comparison that highlights the lack of race and ethnicity detail in the EHR. In addition, the design of EHR fields can hinder this kind of nuanced analysis. In our EHR, when a respondent reports multiple races, the available response option is “multiracial,” which triggers a free-text box for recording the specific races, rather than allowing multiple races to be selected from a preset menu. Converting to a multiselect response design for race and ethnicity would help resolve this issue.

Our findings provide impetus and opportunities for improvement in race and ethnicity data collection and analysis in health care organizations, including (1) updating race and ethnicity questions and related EHR fields/response options to reflect current best practices and (2) adopting the U.S. Census Bureau approach to racial and ethnic grouping for descriptive and comparative analyses. The U.S. Office of Management and Budget sets standards for race and ethnicity data collection and reporting in the United States, and their recently published 2024 rule updates guidance that was last published in 1997.^25,26^ Adopting these updated standards is expected to improve data accuracy and detail. For race and ethnicity data analysis, using “alone,” “in combination,” and “alone or in combination” categories, as in this study, provides a fuller representation of patients, which should lead to more accurate conclusions than can be found in traditional analyses using categories that obscure the identities of those with multiracial, multiethnic, or less commonly listed backgrounds. As disparities analysis becomes standard practice in hospitals, adoption of such new analytical approaches can advance our understanding of where health equity problems lie and how to address them.

Our study has limitations. Because our goal was to determine EHR data accuracy, we designed the survey to capture the gold standard for patient racial and ethnic identity rather than mirror existing EHR questions. A study focused on how consistently the hospital’s processes are implemented would provide important information about whether hospital data collection processes were working as intended. Our team included three surveyors, introducing potential variability in survey delivery. We mitigated this risk through standard surveyor training, a pilot period, and repeated surveyor meetings throughout the study to ensure consistency. Because we used a custom survey and an emerging analysis approach, our findings will not be fully comparable with studies that used different types of gold standard data or traditional race and ethnicity categorization. Finally, we studied only our organization. The wide variability in data collection and storage practices among hospitals and EHRs means that our accuracy outcomes are applicable only to our specific circumstances. Nevertheless, common challenges related to race and ethnicity data collection will likely occur at other hospitals. Using an approach similar to ours can uncover such problems and point toward novel solutions.

## Health Equity Implications

Health equity cannot be achieved without a full understanding of health care experiences across a range of racial and ethnic identities. In the pediatric setting, conventional race and ethnicity data collection and EHR design lead to inconsistent data accuracy, threatening the reliability of disparities analyses and hindering full expression of patient identity. Improved EHR design and new data collection and analysis methods are needed throughout the health care system to overcome the limits of the current race and ethnicity data paradigm, especially related to multiracial/ethnic identity. Without such improvements, racial and ethnic health equity will remain elusive.

## Abbreviations Used

COVID: Coronavirus disease
EHR: Electronic health record
REDCap: Research Electronic Data Capture
SE: Standard Error

## Appendix: Survey Instrument

Note: this study reports on the following subset of questions from a longer survey that included information on language preferences, as well.

### Demographics

Q1. Study Site
  - Urgent Care
  - Inpatient Service
  - Emergency Department
  - Primary Care Clinic

Q2. Patient First and Last Names: ____________
Q3. Patient Age
  - <1 year
  - 1-4 years
  - 5-11 years
  - 12-17 years
  - ≥18 years

Q4. Patient Medical Record Number: _________

### Parent/Guardian 1

Q5. Parent/guardian relationship to patient
  - Mother (include biological mother, stepmother, adoptive mother)
  - Father (includes biological father, stepfather, adoptive father)
  - Parent’s partner
  - Grandmother
  - Grandfather
  - Other legal guardian [free text box]

### Parent/Guardian 2 (if applicable)

Q6. Parent/guardian relationship to patient
  - Mother (include biological mother, stepmother, adoptive mother)
  - Father (includes biological father, stepfather, adoptive father)
  - Parent’s partner
  - Grandmother
  - Grandfather
  - Other legal guardian [free text box]

### Parent/Guardian 3 (if applicable)

Q7. Parent/guardian relationship to patient
  - Mother (include biological mother, stepmother, adoptive mother)
  - Father (includes biological father, stepfather, adoptive father)
  - Parent’s partner
  - Grandmother
  - Grandfather
  - Other legal guardian [free text box]

### Child Race/Ethnicity

*[If patient age <12 years, survey continues with question 8 through 11]*

*[If ≥12 years old, survey continues with questions 12 through]*

Now we’d like to ask a couple of questions about your child’s race and ethnicity.

Q8. Is your child Hispanic or Latino/a?
  - Yes
  - No

*[If “yes,” survey goes to question Q9, if “no,” survey skips to Q10]*

Q9. What specific Hispanic or Latino/a background or backgrounds does your child have? You may give more than one answer.
  *[List of 23 specific backgrounds in survey form, plus “other” with free text box and “prefer not to answer”]*
Q10. What word or words best describe your child’s race or ethnicity? You may give more than one answer.
  *[List of 13 specific races/ethnicities, plus “other” with free text box, “unsure,” and “prefer not to answer”]*
Q11. Are there any other words you would use to describe your child’s heritage, background, or ancestry?
  - Yes [free text box]
  - No

Q12. Do you think of yourself as Hispanic or Latino/a?
  - Yes
  - No

*[If “yes,” survey goes to question Q13, if “no,” survey skips to Q14]*

Q13. What specific Hispanic or Latino/a background or backgrounds do you have? You may give more than one answer.
  *[List of 23 specific backgrounds in survey form, plus “other” with free text box and “prefer not to answer”]*
Q14. What word or words best describe your race or ethnicity? You may give more than one answer.
  *[List of 13 specific races/ethnicities, plus “other” with free text box, “unsure,” and “prefer not to answer”]*
Q15. Are there any other words you would use to describe your heritage, background, or ancestry?
  - Yes [free text box]
  - No

## References

[1] Frey WH. New 2020 Census Results Show Increased Diversity Countering Decade-Long Declines in America’s White and Youth Populations. Brookings Institution; August 13, 2021. Available from: https://www.brookings.edu/articles/new-2020-census-results-show-increased-diversity-countering-decade-long-declines-in-americas-white-and-youth-populations/#:∼:text=Frey%20analysis%20of%202020%20U.S.,and%20Black%20youths%20at%2013.2%25 [Last accessed: February 28, 2024].

[2] Tavernise S, Mzezewa T, Heyward G. Behind the Surprising Jump in Multiracial Americans, Several Theories. New York Times; August 13, 2021. Available from: https://www.nytimes.com/2021/08/13/us/census-multiracial-identity.html [Last accessed: October 22, 2023].

[3] Agency for Healthcare Research and Quality. 2023 National Healthcare Quality and Disparities Report. Available from: https://www.ahrq.gov/research/findings/nhqrdr/nhqdr23/index.html [Last accessed: February 28, 2024]. 38377267

[4] Rodriguez-Lainz A, McDonald M, Fonseca-Ford M, et al. Collection of data on race, ethnicity, language, and nativity by US public health surveillance and monitoring systems: Gaps and opportunities. Public Health Rep 2018;133(1):45–54; doi: 10.1177/0033354917745503 29262290 PMC5805104

[5] Health Research & Educational Trust. A Framework for Stratifying Race, Ethnicity and Language Data. Health Research & Educational Trust: Chicago, IL; 2014.

[6] Cowden JD, Flores G, Chow T, et al. Variability in collection and use of race/ethnicity and language data in 93 pediatric hospitals. J Racial Ethn Health Disparities 2020;7(5):928–936; doi: 10.1007/s40615-020-00716-8 32056162

[7] Mathews K, Phelan J, Jones NA, et al. 2015 National Content Test Race and Ethnicity Analysis Report. US Census Bureau: Washington, DC; February 28, 2017.

[8] Hamilton NS, Edelman D, Weinberger M, et al. Concordance between self-reported race/ethnicity and that recorded in a Veteran Affairs electronic medical record. NC Med J 2009;70(4):296–300. 19835243

[9] Klinger EV, Carlini SV, Gonzalez I, et al. Accuracy of race, ethnicity, and language preference in an electronic health record. J Gen Intern Med 2015;30(6):719–723; doi: 10.1007/s11606-014-3102-8 25527336 PMC4441665

[10] Zingmond DS, Parikh P, Louie R, et al. Improving hospital reporting of patient race and ethnicity–approaches to data auditing. Health Serv Res 2015;50 Suppl 1(Suppl 1):1372–1389; doi: 10.1111/1475-6773.12324 26077950 PMC4545337

[11] Polubriaginof FCG, Ryan P, Salmasian H, et al. Challenges with quality of race and ethnicity data in observational databases. J Am Med Inform Assoc 2019;26(8–9):730–736; doi: 10.1093/jamia/ocz113 31365089 PMC6696496

[12] Proumen R, Connolly H, Debick NA, et al. Assessing the accuracy of electronic health record gender identity and REaL data at an academic medical center. BMC Health Serv Res 2023;23(1):884; doi: 10.1186/s12913-023-09825-6 37608282 PMC10463428

[13] Harris PA, Taylor R, Thielke R, et al. Research electronic data capture (REDCap) – A metadata-driven methodology and workflow process for providing translational research informatics support. J Biomed Inform 2009;42(2):377–381. 18929686 10.1016/j.jbi.2008.08.010 PMC2700030

[14] Tan-McGrory A, Bennett-AbuAyyash C, Gee S, et al. A patient and family data domain collection framework for identifying disparities in pediatrics: Results from the pediatric health equity collaborative. BMC Pediatr 2018;18(1):18; doi: 10.1186/s12887-018-0993-2 29385988 PMC5793421

[15] Institute of Medicine. Race, Ethnicity, and Language Data: Standardization for Health Care Quality Improvement. National Academies Press: Washington, DC; 2009. 25032349

[16] Altman DG. Practical Statistics for Medical Research. Chapman and Hall: Oxford; 1991.

[17] Grilo SA, Santelli JS, Nathanson CA, et al. Social and structural influences on multiracial identification and health: A public health mandate to precisely measure, theorize, and better understand multiracial populations. J Racial Ethn Health Disparities 2023;10(1):427–445; doi: 10.1007/s40615-022-01234-5 35192180

[18] Ho AK, Kteily NS, Chen JM. Introducing the sociopolitical motive × intergroup threat model to understand how monoracial perceivers’ sociopolitical motives influence their categorization of multiracial people. Pers Soc Psychol Rev 2020;24(3):260–286; doi: 10.1177/1088868320917051 32449637

[19] Oh H, Winn JG, Li Verdugo J, et al. Mental health outcomes of multiracial individuals: A systematic review between the years 2016 and 2022. J Affect Disord 2024;347:375–386; doi: 10.1016/j.jad.2023.11.040 38008291

[20] Grilo SA, Santelli JS, Nathanson C, et al. Psychosocial outcomes and peer influences among multiracial adolescents in the United States. Front Public Health 2023;11:852268; doi: 10.3389/fpubh.2023.852268 36923049 PMC10008866

[21] US News & World Report. U.S. News & World Report 2023-2024 Best Hospitals: Health Equity Measures. August 1, 2023. Available from: https://health.usnews.com/media/best-hospitals/Best-Hospitals-Health-Equity-2023-2024 [Last accessed: February 28, 2024].

[22] The Joint Commission. New requirements to Reduce Health Care Disparities. R3 Report – Requirement, Rationale, Reference, Issue 36; June 20, 2022. Available from: https://www.jointcommission.org/-/media/tjc/documents/standards/r3-reports/r3_disparities_july2022-6-20-2022.pdf [Last accessed: February 28, 2024].

[23] Cohn D, Brown A, Lopez MH. Black and Hispanic Americans see their Origins as Central to Who They Are, Less So For White Adults. Pew Research Center; May 14, 2021. Available from: https://www.pewresearch.org/social-trends/2021/05/14/black-and-hispanic-americans-see-their-origins-as-central-to-who-they-are-less-so-for-white-adults/ [Last accessed: November 20, 2023].

[24] Jones N, Marks R, Ramirez R, et al. Improved Race and Ethnicity Measures Reveal U.S. Population is much more Multiracial. US Census Bureau; August 12, 2021. Available from: https://www.census.gov/library/stories/2021/08/improved-race-ethnicity-measures-reveal-united-states-population-much-more-multiracial.html [Last accessed: January 31, 2023].

[25] Office of Management and Budget. Revisions to the Standards for the Classification of Federal Data on Race and Ethnicity. Federal Register 1997;62(210).

[26] Office of Management and Budget. Revisions to OMB’s Statistical Policy Directive No. 15: Standards for Maintaining, Collecting, and Presenting Federal Data on Race and Ethnicity. Federal Register 2024;89(62).

